# Virologic Response at 12 Months Predicts Lower Hepatocellular Carcinoma Risk in Genotype D Chronic Hepatitis B Patients Treated with Nucleos(t)ide Analogues

**DOI:** 10.3390/jcm14082618

**Published:** 2025-04-11

**Authors:** Oguzhan Ozturk, Fatih Guzelbulut, Kamil Ozdil, Huseyin Aykut, Gupse Adalı

**Affiliations:** 1 Department of Gastroenterology, Faculty of Medicine, Biruni University, Gültepe, Halkalı Street Number: 99, Istanbul 34295, Türkiye; kamilozdil@gmail.com; 2 Department of Gastroenterology, Umraniye Training and Research Hospital, University of Health Sciences, Elmalikent, Adem Yavuz Street No:1, Istanbul 34764, Türkiye; f.guzelbulut@gmail.com (F.G.); huseyin.ayk@gmail.com (H.A.); gupseadali@gmail.com (G.A.)

**Keywords:** chronic hepatitis B, hepatocellular carcinoma, virologic response, nucleos(t)ide analogues

## Abstract

**Background/Objectives:** Hepatitis B virus (HBV) is a virus that can cause chronic hepatitis B (CHB) in humans, leading to cirrhosis and hepatocellular carcinoma (HCC). In this study, we aimed to investigate the relationships between early ALT normalization (at 12 months), the virologic response in CHB patients, and the risk of HCC development. **Methods:** Data from a retrospective cohort study involving 616 chronic hepatitis B patients were used. The effects of ALT normalization and virologic response on the risk of developing HCC at 12 months of treatment were analyzed. **Results:** During a median treatment duration of 70.9 months, 36 (5.8%) HCC cases were detected in the total patient population. ALT normalization was detected in 68.83% of patients at 12 months of treatment. The rate of HCC in the group with early ALT normalization was lower than that in the group without ALT normalization, but this difference was not statistically significant (5% vs. 7.8%, *p* = 0.161). At the end of 12 months of treatment, virologic response was detected in 80.68% of the patients. The rate of patients developing HCC was significantly lower in the virologic response group (4.8% vs. 10.1%, *p* = 0.028). However, the risk of developing HCC was also significantly higher in the virologically unresponsive group, according to the virologic response at 12 months (*p* = 0.034). **Conclusions:** According to the results of this study, achieving virologic response at the end of 12 months in genotype D CHB patients treated with nucleos(t)ide analogs (NAs) reduces the risk of developing HCC.

## 1. Introduction

Hepatitis B virus (HBV) is a hepatotropic virus that causes chronic hepatitis in humans and leads to liver-related complications. According to the World Health Organization (WHO), it is estimated that 296 million people have chronic HBV infection [1,2]. Patients with chronic hepatitis B (CHB) may develop serious complications, especially cirrhosis and hepatocellular carcinoma (HCC). The nucleos(t)ide analogs (NAs) entecavir (ETV), tenofovir disoproxil fumarate (TDF), and tenofovir alafenamide (TAF), which are potent antiviral agents, are currently used in the treatment of CHB. These treatments aim to improve necroinflammation in the liver by promoting a virologic and biochemical response. Studies have shown that the alanine aminotransferase (ALT) value, which is considered an indicator of biochemical response, is associated with necroinflammation [3,4].

To date, studies have shown that early ALT normalization (before 1 year) during CHB treatment reduces the risk of HCC and mortality due to hepatic events [5,6]. However, the reasons for this relationship have not been fully elucidated. Different studies have also shown that virologic factors are associated with a risk of HCC development. In particular, it has been shown that high viral load before treatment and failure to maintain virologic response at the end of treatment play important roles in increasing the risk of HCC [7].

In this study, we aimed to investigate the relationships between early ALT normalization (at 12 months), the virologic response in CHB patients, and the risk of HCC development.

## 2. Materials and Methods

Data (printed patient medical follow-up files and all electronic medical documents) of treatment-naïve chronic hepatitis B patients who were started on LAM, ETV, or TDF treatment in two training and research hospitals in Istanbul (Haydarpaşa Numune Training and Research Hospital and Umraniye Training and Research Hospital affiliated with the University of Health Sciences) between January 2007 and December 2021 were collected retrospectively.

Initial laboratory tests and radiological imaging (Ultrasound, USG; Computed Tomography, CT; or Magnetic resonance imaging, MRI) of patients who were 18 years of age or older, admitted to the hospital, and whose HBsAg positivity persisted for more than 6 months were evaluated. Data were evaluated for the necessity of liver biopsy before starting antiviral treatment. Liver biopsy was performed within the scope of the reimbursement conditions of the Social Security Institution for patients other than those diagnosed with cirrhosis clinically, laboratory, and radiologically; pregnant women; and those with coagulopathy that would prevent liver biopsy. Patients who met the criteria for starting antiviral therapy were started on treatment and both electronic and written (printed) files were opened in the Hepatology Clinic. Patients were followed up for at least 1 year through face-to-face control visits every 3 months. The files of patients who started antiviral therapy were reviewed retrospectively in terms of laboratory, radiological imaging, and HCC development.

Exclusion criteria:

1. <18 years;
2. Those diagnosed with HCC at the time of diagnosis or within 1 year of starting treatment;
3. Those without baseline HBV DNA and ALT values;
4. Follow-up < 1 year;
5. Those who have previously received Pegylated interferon treatment
6. Those with HCV, HDV, HIV coinfection.

Hepatitis B surface antigen (HBsAg), hepatitis B envelope antigen (HBeAg), hepatitis B surface antibody (AntiHBs), and anti-hepatitis B envelope antigen (AntiHBe) were measured as s/co (sample signal/cutoff) ratio via an electrochemiluminescent method using the Modular Analitics E170 (Roche Diagnostics) automated system.

HBV DNA levels were measured (as IU/mL) via real-time polymerase chain reaction using the Cobas TaqMan test (Roche Diagnostics). As almost all chronic HBV patients in our country have genotype-D, no additional test was performed to determine the genotype, and the patients were accepted as genotype-D [8,9].

The recommendation of the American Association for the Study of Liver Diseases was adopted as the criteria. According to these criteria, a serum hepatitis B virus DNA of <15 IU/mL was considered a virologic response, and a normal ALT of ≤35 U/L (men) and ≤25 U/L (women) was considered a biochemical response [3].

Accordingly, in all patients with chronic hepatitis B without cirrhosis, whether HBeAg positive or negative, if HBV-DNA was 2000 IU/mL and above, histological activity index (HAI) ≥ 6 or fibrosis ≥ 2 was requested in liver biopsy, and in patients with cirrhosis, only HBV-DNA positivity was requested sufficient [10,11].

In the initial periods of the study, only three patients were started on LAM treatment in line with the Social Security Institution’s policy of covering drug costs. In the subsequent period, after a new arrangement was made in the drug payment policy, entecavir and tenofovir treatment were used in the patients. Additionally, none of our patients received ETV/TDF combined treatment. Lamivudine 100 mg/day, tenofovir 245 mg/day, Entecavir 0.5–1 mg/day doses were used.

The diagnostic criteria of the EASL guideline were used for the diagnosis of HCC. In patients with elevated alpha-fetoprotein (AFP) and/or a mass detected in the liver on ultrasonography (USG), radiological multiphasic CT and dynamic contrast-enhanced MRI were used to diagnose HCC with characteristic features. In liver masses that did not provide characteristic features on CT and MRI or in patients who could not undergo CT/MRI for any reason, a mass biopsy was performed to diagnose the disease [12].

Obesity was defined according to the World Health Organization. According to the World Health Organization, obesity in adults (over 18 years of age) is defined as a body mass index (BMI) of 30 kg/m^2^ and above, and a BMI between 25 and 29.9 kg/m^2^ is called overweight (pre-obesity) [13].

Diabetes was defined as HbA1c  ≥ 6.5% (48 mmol/mol) or FPG ≥ 7.0 mmol/L (126 mg/dL) or 2 h plasma glucose ≥ 11.1 mmol/L (200 mg/dL) during an OGTT or in a patient with classic symptoms of hyperglycemia or hyperglycemic crisis, a random plasma glucose ≥ 11.1 mmol/L (200 mg/dL) [14].

Fatty liver is a general term that refers to a population in which macrovesicular hepatic steatosis is seen in individuals in whom ≥ 5% of hepatocytes exhibit macrovesicular steatosis and who consume little or no alcohol (defined as <20 g/day for women and <30 g/day for men) [15]. Whether or not our patients had fatty liver was determined based solely on liver biopsy data. Those without biopsy data were excluded from evaluation for fatty liver.

## 3. Statistics

Values are presented as means, medians, or numbers of patients (%). The cumulative incidence of HCC was calculated using the Kaplan–Meier method. To identify the risk factors for predicting HCC, univariate and multivariate analyses were performed through Cox regression analysis, including clinical variables at baseline. In univariate analysis, if a variable satisfied *p* < 0.250, that variable was used for multivariate analysis. Statistical significance was set at *p* < 0.05. Statistical analyses were conducted using IBM^®^ SPSS^®^ Statistics Version 29.0.1. All reported *p*-values are two-sided, and *p* < 0.05 was considered statistically significant.

## 4. Results

**Figure 1:**
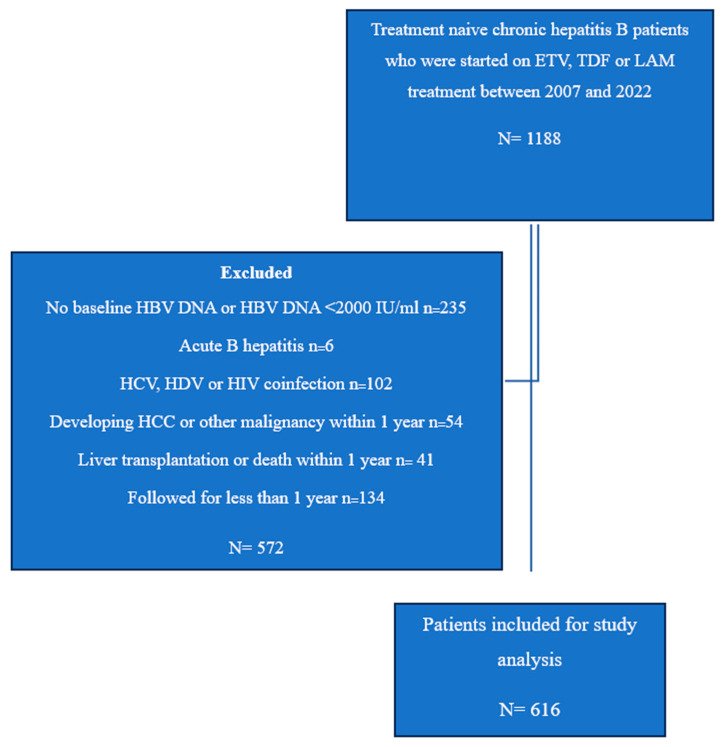
Study flow chart. HBV, hepatitis B virus; HCV, hepatitis C virus; HDV, hepatitis D virus; HIV, human immunodeficiency virus; HCC, hepatocellular carcinoma; LAM, lamivudine; TDF, tenofovir disoproxil fumarate; ETV, entecavir.

Data from 1188 patients were evaluated. After excluding patients who were not eligible for the study, a total of 616 patients were included in the study (see Figure 1 for the study flow chart), comprising treatment-naïve CHB patients who were treated with NAs for more than 1 year from 2007 to 2022. All patients were without a history of HCC (Table 1).

Of all patients, 64.3% were male, and the median age was 49.5 years. The median HBV DNA level was 1,022,398 IU/mL, and 25.3% of patients had cirrhosis. The median ALT level was 55 IU/L, and the median follow-up time was 70.9 months. During the follow-up period, 36 (5.8%) HCC cases were detected in the total patient population.

The ALT normalization group was defined as the group that continued to have normal ALT after starting treatment and had normal ALT at the end of treatment. ALT normalization was detected in 68.83% of patients at 12 months of treatment. When patients were grouped according to ALT normalization at the 12th month of treatment (early ALT normalization), the median age was higher in the ALT-normalized group (50.65 vs. 48.3). While obesity and diabetes were similar in both groups, HBV DNA levels, median ALT and cirrhosis rates were higher in the ALT-elevated group (Table 2).

At the end of 12 months of treatment, the median HBV-DNA level in the normal ALT group was significantly lower, while the virological response rate was also significantly higher.

At the end of 12 months of treatment, virologic response was detected in 80.68% of the patients. When patients were grouped according to virologic response at 12 months of treatment, the median age was higher in the virologic response group (51.2 years vs. 42.4 years). In the group without virologic response, the median HBV DNA and ALT levels were significantly higher (both *p* < 0.001). Platelet count and cirrhosis rate were similar in both groups. Again, the number of patients showing ALT normalization at the end of the first year was significantly higher in the virologic response group (72.3% vs. 55.9%, *p* < 0.001). The rate of patients developing HCC was also significantly lower in the virologic response group (4.8% vs. 10.1%, *p* = 0.028; Table 3).

### 4.1. Survival According to ALT Normalization and Virologic Response

**Figure 2:**
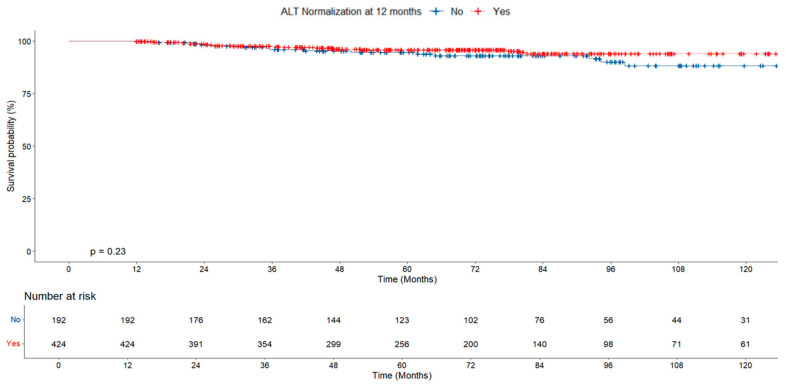
Survival probability according to ALT normalization at 12 months.

**Figure 3:**
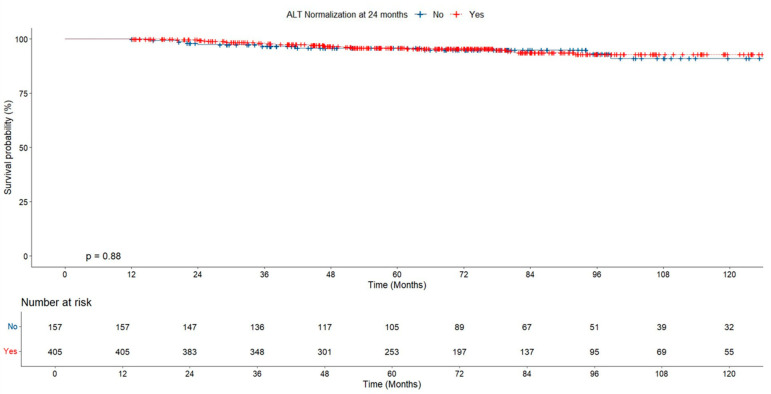
Survival probability according to ALT normalization at 24 months.

Cumulative survival was similar in groups with and without early ALT normalization (at 12 months). Similarly, no significant difference was found between groups with and without ALT normalization at 24 months (Figure 2 and Figure 3).

**Figure 4:**
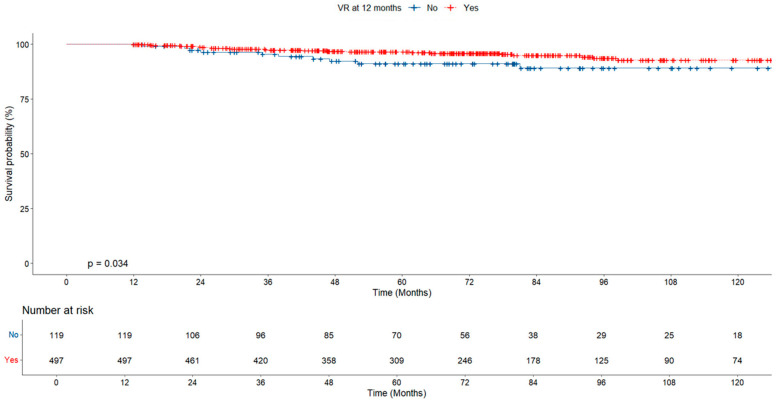
Survival probability according to virologic response at 12 months.

**Figure 5:**
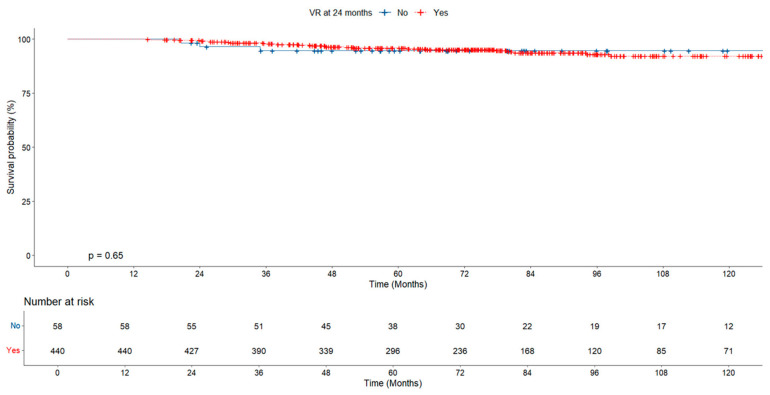
Survival probability according to virologic response at 24 months.

In the evaluation of cumulative survival according to virologic response, survival was found to be significantly higher in the group showing early virologic response (at 12 months) than in those without response, while no significant difference was found between the groups at 24 months (Figure 4 and Figure 5).

### 4.2. HCC Development According to ALT Normalization and Virologic Response

**Figure 6:**
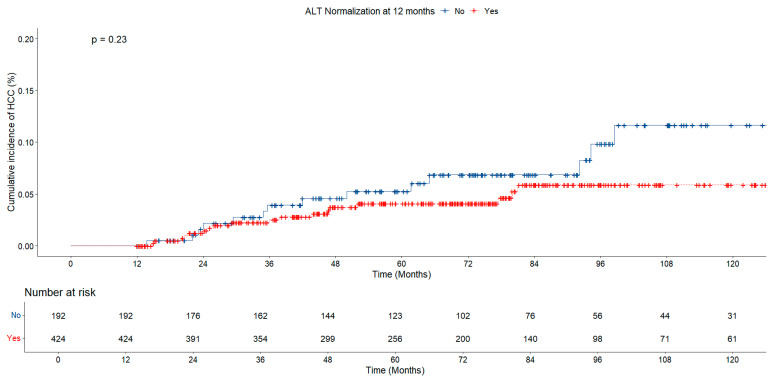
Risk of HCC according to ALT normalization at 12 months in chronic hepatitis B patients.

During a median follow-up of 5.9 years, 36 (5.8%) patients developed HCC. All patients with HCC were treated in accordance with the Modified Barcelona-Clinic Liver Cancer (BCLC) staging system and treatment strategy [12]. When the patients were evaluated in terms of HCC development, the HCC rate in the group with early ALT normalization was found to be lower than in the ALT non-normalized group, but no statistically significant difference was found between the groups (5% vs. 7.8% *p* = 0.161; Table 2). No significant difference was found in the risk of HCC based on ALT normalization at 12 months in both groups (*p* = 0.23, Figure 6).

**Figure 7:**
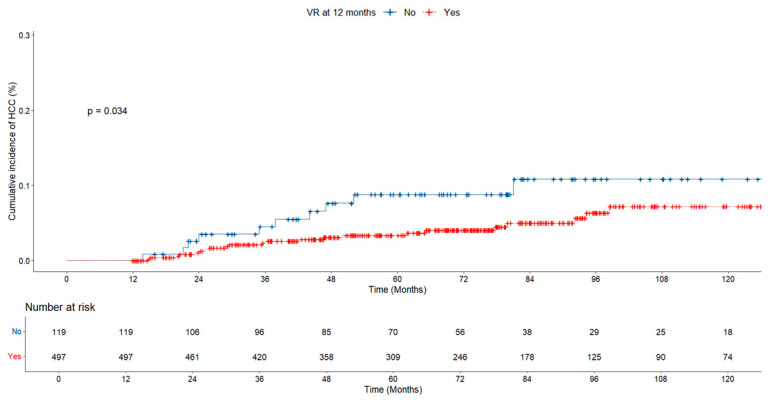
Risk of HCC according to virologic response at 12 months in chronic hepatitis B patients.

When HCC development was evaluated according to virologic response, the HCC rate in the group without virologic response at 12 months was found to be significantly higher than in the group with virologic response (10.1% vs. 4.8%, *p* = 0.028; Table 3). According to virological response at 12 months, the risk of developing HCC was also significantly higher in the group without virological response (*p* = 0.034; Figure 7).

Due to the missing data for fatty liver and BMI, they were not included in the multivariate analysis. In the multivariate analysis, the independent predictors of HCC development were virologic response at month 12, male sex, baseline cirrhosis, and age (Table 4).

## 5. Discussion

Chronic hepatitis B infection (CHB) is an important cause of cirrhosis and hepatocellular carcinoma (HCC) in underdeveloped and developing countries. With widespread vaccination and the introduction of potent antiviral drugs, the development of HBV-associated cirrhosis and HCC is decreasing worldwide. However, it has been observed that hepatic events (e.g., cirrhosis, varicose veins, and ascites) and HCC may develop in CHB patients, even during antiviral therapy. Therefore, the factors affecting the development of hepatic events and HCC are still under investigation.

Wong V.W. et al. have prospectively investigated liver-related events (hepatocellular carcinoma, ascites, spontaneous bacterial peritonitis, variceal bleeding, liver transplantation, and death) in patients with chronic hepatitis B through four randomized controlled trials involving 195 patients. In their study, it was determined that biochemical (ALT normalization) and histological responses achieved in patients with CHB were associated with regression of cirrhosis and reduction of liver-related complications [16].

Recent studies have shown that achieving ALT normalization within the first year of antiviral therapy (i.e., early ALT normalization) is associated with a lower incidence of HCC and other hepatic events. The study conducted by Kim S. et al. analyzed 610 CHB patients treated with entecavir or tenofovir disoproxil fumarate, and it was found that patients with ALT normalization within 1 year had a significantly lower cumulative HCC incidence than those with non-normalized ALT (*p* < 0.001) [17].

Supporting this finding, another study analyzed 4639 CHB patients and found that early ALT normalization was associated with a lower risk of HCC, independent of fatty liver, cirrhosis, and virologic response during treatment [6]. In a large-scale study conducted on this subject, 21,182 CHB patients receiving antiviral treatment were examined, and it was shown that the risk of developing HCC was significantly reduced in patients with early ALT normalization. Furthermore, other hepatic events—such as cirrhosis, variceal bleeding, hepatic encephalopathy, and ascites—were also detected less in patients with early ALT normalization [5].

In our study, it was found that patients with early ALT normalization developed HCC at a lower rate than those with non-normalized ALT. However, no statistically significant difference was found (5% vs. 7%, *p* = 0.161). The fact that the number of patients was not sufficient to make the statistical data strong may be a factor in this regard. However, as is well-known, studies have shown that host (patient) characteristics and viral characteristics may lead to differences in the development of HCC, regardless of ALT normalization, including viral genotypic characteristics. Studies have shown that individuals infected with genotype C and D HBV virus are more likely to become chronic and develop HCC than those with other genotypes. In addition, it has been shown that the disease is more severe, and ALT elevation is more common in patients with genotype C CHB [18].

Many studies have reported that different genotypes and subgenotypes show different geographical distributions and are associated with disease progression, clinical progression, response to antiviral treatment, and prognosis [8]. The above-mentioned studies were conducted in countries where genotypes B and C are dominant. In our country, almost all CHB patients have genotype D HBV virus and the potential for developing HCC is higher than other genotypes, such as genotype C [8]. In our study, no significant difference was found in terms of HCC development between the ALT-normalized and non-ALT-normalized groups. This result suggests that viral genotypic characteristics also play a role in the development of HCC, independent of ALT normalization.

ALT normalization has also been shown to be associated with different viral genomic features in some studies. According to one study, novel biomarkers such as pre-genomic HBV RNA (pgRNA) and quantitative HBV surface antigen (qHBsAg) showed significant correlations with changes in ALT levels. In particular, pgRNA was found to be a better predictor of ALT changes than HBV DNA in patients treated with NAs [19]. Furthermore, there have been reports that the presence of another viral genomic feature—the mutated large hepatitis B surface—may have an effect on late ALT normalization and HCC carcinogenesis [20]. As shown in these studies, different viral genomic features may also affect the ALT normalization results. Since viral genomic features such as these were not investigated in the population in both the above-mentioned studies and our study, the effect of these factors on ALT normalization could not be evaluated. Studies with larger numbers of participants are needed for elaboration of this subject.

Failure to achieve ALT normalization may also be related to host factors. Cirrhosis is a well-known host factor in the development of HCC. In the study by Kim et al., the rate of cirrhosis in the elevated ALT group was approximately 45.5%, while it was half that rate in the normal ALT group. In our study, the cirrhosis rate in the elevated ALT group was 30%, and in the normal ALT group was 22.6%. Accordingly, the higher rate of cirrhosis patients in the high ALT group in the Kim et al. study was thought to contribute to the development of HCC at a higher rate in this group.

Some studies investigating the association between early ALT normalization and HCC have shown that high BMI (BMI > 25) and/or the presence of fatty liver are positively associated with high ALT levels and the risk of developing HCC after 1 year of treatment. However, it has been observed that the probability of ALT normalization is decreased in patients with metabolic syndrome risk factors [5,17,21,22]. In the study conducted by Kim et al., the BMI rate was significantly higher in the elevated ALT group than in the normal ALT group, while in the study conducted by Wong et al., diabetes and fatty liver patients were found to be higher in the elevated ALT group. However, in our study, diabetes, high BMI and fatty liver rates were found to be at similar levels in the ALT normalized and elevated ALT groups. When examined according to ALT normalization, it is thought that these results may have contributed to the detection of HCC at similar rates in both groups.

In conclusion, many viral factors such as HBV genotype, specific viral mutations, as well as host factors (fatty liver, presence of cirrhosis, etc.) may affect ALT normalization after treatment. These differences in the treatment population in the studies mentioned above and in our study may explain the differences in ALT normalization after treatment. Extensive research is needed on this subject.

In our study, the independent predictors of HCC development in the multivariate analysis were virologic response at month 12, male sex, baseline cirrhosis, and age. Studies have shown that the presence of cirrhosis, advanced age, and male sex are well-known risk factors for the development of HCC [23,24].

Achieving a virologic response with potent antiviral agents is one of the main goals in the treatment of CHB, as studies have shown that the development of cirrhosis and HCC decreases in patients who achieve a virologic response. One study has evaluated data from a cohort of 1447 CHB patients treated with entecavir and demonstrated that the risk of developing HCC increased when virologic response was not maintained [25]. Another study comparing the potent antiviral agents ETV and TDF has emphasized the importance of maintenance of the viral response. A total of 1794 naïve CHB patients were evaluated in this study. When the viral response was maintained with both drugs, no significant difference was found between the groups in terms of HCC development, death, or transplantation [26].

Another prospective cohort study including 3653 participants showed that high serum HBV DNA level (≥10,000 copies a/mL) was a strong risk predictor of hepatocellular carcinoma, independent of HBeAg, serum alanine aminotransferase level, and liver cirrhosis. In this study, participants with persistently elevated serum HBV DNA levels during follow-up had the highest risk of hepatocellular carcinoma [27].

In our study, the rate of hepatocellular carcinoma was significantly higher in the group without virologic response than in the group with virologic response at the 12th month of treatment (10.1% vs. 4.8%, *p* = 0.028). The baseline HBV DNA level was significantly lower in the group with virologic response. However, both groups were similar in terms of obesity, fatty liver, and cirrhosis. The risk of developing HCC was also significantly higher in the group with no virologic response (*p* = 0.034; Figure 5). Our findings are consistent with those of the large-scale studies mentioned above [25,26,27].

When virologic response was achieved at the end of the 24th month, no significant difference was found between the groups in terms of HCC development. This may be related to the late achievement of a sustainable viral response. The low rate of HCC development in individuals who achieved virologic response at the end of the 12th month in our study also supports this idea. Similarly, one study examined 4639 CHB patients treated with NAs and showed that virologic response at 24 months did not reduce the risk of HCC [6]. One of the important factors affecting the sustained viral response is the pretreatment HBV DNA level. In one study, high HBV DNA load, high HBsAg quantification, and positive HBeAg results were shown to reduce the sustained virologic response and increase the risk of low viremia in patients treated with entecavir [28]. As a result, high baseline HBV DNA levels negatively affect the early virologic response. The persistence of HBV DNA positivity, even in the form of low viremia, may also activate oncogenic cascades. Therefore, it can be considered that the establishment of a sustainable virologic response at an early stage may prevent the development of oncogenic cascades [29,30].

The first limitation of this study is its retrospective nature. Accordingly, our fatty liver data were incomplete. However, the data was only derived from patients with clear evidence of fatty liver disease on liver biopsy. In order to ensure the reliability of the data, the data of patients who did not provide clear information about steatosis in their liver biopsy —which constituted approximately one-third of all patients—were not included in the analysis. However, the data for the study cohort were mostly generated during face-to-face visits with the patient and by expert gastroenterologists. Therefore, regular use of NAs was ensured, and problems such as medication non-compliance were minimized. Secondly, our study population was smaller than those of large-scale studies in the literature. Third, as CHB patients in our country are predominantly genotype D, we did not have the opportunity to compare the effects of virologic response with respect to other genotypes.

## 6. Conclusions

In our study, it was found that achieving virologic response at the end of 12 months in genotype D CHB patients treated with NAs reduced the risk of developing HCC. In this sense, we believe that delayed viral response may increase the risk of developing HCC. Therefore, achieving early virologic response in CHB patients should be one of the main goals. Additionally, comprehensive studies are needed to examine both viral and host factors affecting the immunopathogenesis of CHB-related HCC.

## Figures and Tables

**Table 1:** Baseline characteristics of patients.

	n = 616
Age, years, median (min–max)	49.5 (18–93)
Gender, male, n (%)	396 (64.3)
BMI *, kg/m^2^, median (min–max)	25.63 (16.94–47.80)
Obesity, n (%)	58 (20.2)
Diabetes, n (%)	99 (16.2)
Fatty liver ^†^, n (%)	231 (60)
HBV DNA, IU/mL, median (min–max)	1,022,398 (40–2,397,000,000)
Creatinine, mg/dL, median (min–max)	0.79 (0.33–4.74)
AST, IU/L, median (min–max)	39 (10–1650)
ALT, IU/L, median (min–max)	55 (7–1556)
Albumin, g/dL, median (min–max)	4.1 (1.8–5.3)
Total bilirubin, mg/dL, median (min–max)	0.75 (0.16–32.85)
Platelets, 1000/mm^3^, mean (SD)	194.2 ± 67.4
Cirrhosis, n (%)	156 (25.3)
ETV/TDF/LAM, n (%)	228/383/3 (37/62.2/0.5)
Follow-up time, months, median (min–max)	70.9 (12–187)

BMI, body mass index; HBV, hepatitis B virus; ALT, alanine aminotransferase; AST, aspartate aminotransferase; ETV, entecavir; TDF, tenofovir disoproxil fumarate; LAM, lamivudine; * information was unavailable for 329 patients. ^†^ Fatty liver information was obtained from liver biopsy data but was not available for 231 patients.

**Table 2:** Comparison of baseline characteristics of patients with and without ALT normalization at 12 months.

	Patients with Elevated ALT at 12 Months (n = 192)	Patients with Normal ALT at 12 Months (n = 424)	*p*
Age, years, mean ± SD	48.30 ± 12.9	50.65 ± 14.1	0.05
Gender, male, n (%)	123 (64.1)	273 (64.4)	0.938
BMI *, kg/m^2^, median (IQR)	26 (5.9)	25.7 (6)	0.319
Obesity, n (%)	15 (17.9)	43 (21.2)	0.523
Diabetes, n (%)	36 (18.9)	63 (15)	0.221
Fatty liver ^†^, n (%)	70 (61.9)	161 (59.2)	0.615
HBV DNA, IU/mL, baseline, median (IQR)	1,815,000 (19,944,796)	633,874 (17,112,132)	0.053
Creatinine, mg/dL, median (IQR)	0.79 (0.15)	0.79 (0.21)	0.518
AST, IU/, median (IQR)	42 (37)	37 (46)	0.009
ALT, IU/L, median (IQR)	68 (70)	48 (82)	<0.001
Albumin, g/dL, median (IQR)	4.1 (0.7)	4.2 (0.6)	0.052
Total bilirubin, mg/dL, median (IQR)	0.72 (0.56)	0.77 (0.55)	0.896
Platelets, 1000/mm^3^, mean ± SD	197.1 ± 65.4	192.8 ± 68.3	0.465
Cirrhosis, n (%)	60 (31.3)	96 (22.6)	0.023
ETV/Tenofovir, n (%)	59 (31.2)/130 (68.8)	169 (39.9)/255 (60.1)/0	0.041
ALT normalization on month 24, n (%)	70 (39.3)	335 (87.2)	<0.001
HBV DNA on month 12, median (IQR)	39 (18)	20 (9)	<0.001
Virologic response on month 12, n (%)	139 (72.4)	358 (84.4)	<0.001
HBV DNA on month 24, median (IQR)	37 (24)	20 (10)	<0.001
Virologic response on month 24, n (%)	127 (81.9)	313 (91.3)	0.003
Follow-up time, months, median (IQR)	73.6 (53)	69.8 (48)	0.184
ALT on month 12, median (IQR)	41 (22)	20 (10)	<0.001
ALT on month 24, median (IQR)	34 (24)	20 (10)	<0.001
HCC, n (%)	15 (7.8)	21 (5)	0.161

BMI, body mass index; HBV, hepatitis B virus; HCC, hepatocellular carcinoma; ALT, alanine aminotransferase; AST, aspartate aminotransferase; LAM, lamivudine; TDF, tenofovir disoproxil fumarate; ETV, entecavir. * information was unavailable for 329 patients. ^†^ Fatty liver information was obtained from liver biopsy data but was not available for 231 patients.

**Table 3:** Characteristics of patients according to virologic response (VR).

	With VR 12 (n = 497)	Without VR 12 (n = 119)	*p*
Age, years, median (IQR)	51.2 (19)	42.4 (22)	<0.001
Gender, male, n (%)	313 (63)	83 (69.7)	0.166
BMI *, kg/m^2^, median (IQR)	25.8 (5.8)	24.9 (5.9)	0.076
Obesity, n (%)	49 (21.7)	9 (14.8)	0.232
Diabetes, n (%)	83 (16.8)	16 (13.7)	0.405
Fatty liver ^†^, n (%)	197 (60.8)	34 (55.7)	0.459
HBV DNA, IU/mL, baseline, median (IQR)	330,851 (6,059,380)	20,000,000 (40,560,712)	<0.001
Creatinine, mg/dL, median (IQR)	0.79 (0.55–2.70)	0.78 (0.41–1.04)	0.054
AST, IU/L, median (IQR)	36 (39)	52 (60)	<0.001
ALT, IU/L, median (IQR)	48 (69)	88 (95)	<0.001
Albumin, g/dL, median (IQR)	4.2 (0.6)	4.1 (0.7)	0.102
Total bilirubin, mg/dL, median (IQR)	0.74 (0.56)	0.77 (0.49)	0.612
Platelets, 1000/mm^3^	192 ± 67	184 ± 65	0.397
Cirrhosis, n (%)	126 (25.4)	30 (25.2)	0.974
ETV/Tenofovir/LAM, n (%)	187/307/3 (37.6/61.8/0.6)	41/78/0 (34.5/65.5/0)	0.631
ALT normalization on month 12, n (%)	350 (72.3)	66 (55.9)	<0.001
ALT normalization on month 24, n (%)	347 (76.1)	58 (54.7)	<0.001
HBV DNA on month 12, median (IQR)	0 (0)	136 (1901)	<0.001
HBV DNA on month 24, median (IQR)	0 (0)	0 (68)	<0.001
Follow-up time, months	71 (49)	68 (52)	0.649
ALT on month 12, median (IQR)	24 (17)	29 (24)	<0.001
ALT on month 24, median (IQR)	22 (15)	28 (21)	<0.001
HCC, n (%)	24 (4.8)	12 (10.1)	0.028

BMI, body mass index; HBV, hepatitis B virus; HCC, hepatocellular carcinoma; ALT, alanine aminotransferase; AST, aspartate aminotransferase; LAM, lamivudine; TDF, tenofovir disoproxil fumarate; ETV, entecavir. * information was unavailable for 329 patients. ^†^ Fatty liver information was obtained from liver biopsy data but was not available for 231 patients.

**Table 4:** Univariate and multivariate Cox regression analyses for predictors of HCC risk in patients with chronic hepatitis B.

	Univariate Analysis		Multivariate Analysis	
Variables	HR (95% CI)	*p*-Value	HR (95% CI)	*p*-Value
Baseline HBV DNA	1.00 (1.00–1.00)	0.087	-	-
Fatty liver	0.53 (0.24–1.15)	0.110	-	-
ALT normalization at 12 months	0.66 (0.39–1.39)	0.236	-	-
Virologic response 12 months	0.48 (0.24–0.96)	0.038	0.26 (0.08–0.86)	0.027
Gender (male/female)	4.37 (1.54–12.3)	0.005	-	-
Diabetes (yes/no)	1.73 (0.81–3.69)	0.153	-	-
BMI	1.14 (1.04–1.24)	0.004	-	-
Baseline cirrhosis	8.53 (4.11–17.70)	<0.001	3.26 (1.05–10.10)	0.04
Age	1.08 (1.05–1.10)	<0.001	1.05 (1.00–1.10)	0.02

BMI, body mass index; HCC, hepatocellular carcinoma; ALT, alanine aminotransferase. In univariate analysis, virologic response at month 12, male sex, BMI, baseline cirrhosis, and age were associated with HCC development.

## Data Availability

The datasets used and analyzed during the current study are available from the corresponding author upon reasonable request.
